# Farther Account of a New Method of Treating Diseases of the Joints of the Knee and Elbow

**Published:** 1790

**Authors:** H. Park

**Affiliations:** one of the Surgeons of the Liverpool Infirmary


### III. *Farther Account of a new Method of treating Diseases of the Joints of the Knee and Elbow. Communicated in a Letter to Dr.* Simmons *by Mr.* H. Park, *one of the Surgeons of the Liverpool Infirmary.*

IN the year 1783 I ventured to obtrude on the world a ſmall pamphlet, pointing out a mode of treating ſome of the affections of the large articulations, which I then believed had not been attempted by any other practitioner. To the hiſtory of the caſe of Hector M'Caghen, there related, I have now to add, that he afterwards made ſeveral voyages to ſea; in which he was able to go aloft with conſiderable agility, and to perform all the duties of a ſeaman: that he was twice ſhipwrecked, and ſuffered great hardſhips, without feeling any farther complaint in that limb; but was at laſt unfortunately drowned by the overſetting of a flat in the river Merſey.

As the propriety, however, of adopting ſuch a practice can only be determined by a number of experiments, I feel myſelf equally bound to communicate to the Public the event of my ſecond attempt of this nature, although it proved as unfortunate as the firſt was ſucceſsful.

The

The ſubject of this operation was Charles Harriſon, aged thirty years, by trade a wheel-wright, and to appearance a ſtrong, robuſt man; but who was, as I afterwards learned, of a highly ſcrophulous family.

His knee, which had been diſeaſed about three years, was more enlarged than that of Hector M'Caghen; the diſeaſe in the ſoft parts was more extenſive; and a conſiderable abſceſs had formed, which extended ſome inches below the joint, on the outſide of the leg, but had not yet opened.

The operation was peformed, agreeably to the patient's choice, on the 22d of June, with little variation, as to the *modus operandi,* from the former one, except that an opening was firſt made into the abſceſs, as well to anſwer the purpoſe of a depending drain afterwards, as to afford an opportunity of examining the ſtate of the fibula, which was not found diſeaſed.

Two ſmall arteries were taken up, and the cavity lightly filled with lint. An anodyne was given immediately after the operation; notwithſtanding which he paſſed the day in a good deal of pain; but by repeating the opiate in the evening had an eaſy night.

The wound was not opened till the 27th, when only the bandages and external dreffings, which were becoming offenfive, were changed, and he was removed into a frefh bed. The knee appeared large, but the leg and thigh were pretty free from tenfion; the difcharge was moderate in quantity, and good in quality, coming moftly from the depending opening; and the man feemed very well in his general health.

The dreffings did not all come out till July the 1ft, when the whole furface of the wound looked clean, and the granulations were fo luxuriant, that the ends of the bones were covered, and the cavity was in a great meafure filled up.

July 8th. The difcharge was much diminifhed, very little coming from the depending opening.

—— 20th. Some degree of union appeared to have taken place; the bulk of the knee and the furface of the wound were diminifhing apace. Some pus, however, was preffed out of the granulations, as from a fponge.

—— 24th. He had a diarrhœa during the two laft days. A fmall quantity of pus had made its way through the cicatrix of an old iſſue below the inſide of the knee, but no more could be preſſed out of the granulations. The union appeared to be gaining ſtrength.

July 31ſt. He had gotten the limb into a bad poſition; the union was a good deal looſened, and there was rather more diſcharge; the bulk of the limb and ſurface of the wound were diminiſhing faſt; the diarrhœa had ceaſed, but he had night ſweats, looked ill, felt languid, and complained of a good deal of pain in the other leg, which was ſwollen and œdematous.

Auguſt 10th. The affection of the other leg was much abated.

——— 21ſt. The ſweats had ceaſed, and his ſtrength was much improved. The diſcharge was ſmall, and the union apparently ſtronger than it had yet been. We now began to take him out of bed frequently.

——— 26th. He complained of more pain, ſwelling, and ſoreneſs. The union appeared more looſe, and the diſcharge more conſiderable; but he ſeemed to be in tolerably good general health. About this time I learnt that his family were highly ſcrophulous.

September 7th. The diſcharge was ſtill conſiderable. He had been languid, and had but little appetite during the two laſt days; but ſeemed better to-day.

September 14th. The diſcharge was again become moderate, with appearance of more union; and the healing of the wound was advancing.

——— 30th. He had a troubleſome diarrhœa.

October 3d. He had inceſſant bilious vomiting and purging, with ſevere griping, great internal heat, and troubleſome aphthæ. By theſe complaints he was ſuddenly brought into ſuch a ſtate as to preclude every idea of removing the limb, could we have had ever ſo much reaſon to hope that, by ſo doing, we ſhould have removed the whole diſeaſe. In this ſtate he continued until the 13th, and then ſunk in ſpite of all our efforts.

Soon after the publication of my little pamphlet, the late Mr. Filkin, of Northwich, informed Dr. Binns, of this town, that he had performed a ſimilar operation, about twenty years before, with ſucceſs. The Doctor, at my requeſt, applied to Mr. Filkin for the particulars of the caſe, but was diſappointed in his attempts to obtain them; that gentleman being ſoon after ſeized with a paralytic affection, which greatly impaired his faculties, and at laſt terminated in his death. I have been, however, ſince favoured with a letter from his ſon, at preſent ſurgeon in Northwich; of which the following is an extract:

"You will, I fear, think me very remiſs in "not anſwering your kind favour long before; "but as my father's notes do not deſcribe the "caſe of the operation of the knee ſo plainly "as I could wiſh, I have waited till an oppor- "tunity occurred when I could ſee the man, to "have what he knew on the matter; and "though all I can collect on the ſubject is very "trifling, ſtill I beg leave to ſend you what "little information I have gained.

"The patient was always of a ſcrophulous "habit, and had for many years a tumour on "the knee, which gradually increaſed in ſize, "and to which every topical application was "uſed without effect. By accident, falling "from a horſe, the patella was fractured, and "from a ſmall wound there was diſcharged "about half a pound of fœtid, foul-coloured "pus: amputation was immediately propoſed; "but the parents not conſenting, my father "was called in. Having frequently thought

" this method might fometimes fucceed, and
" having performed it once on the dead body,
" he propofed it to the parents of the patient
" in this cafe, though it was an unfavourable
" one, the patient's general health being much
" impaired. The parents confenting, a day
" was fixed for the operation, which was per-
" formed on the 23d of Auguft, 1762. The
" ligaments were found in a very floughy,
" fuppurative ftate, with the cartilages greatly
" injured, and the heads of the bones much
" difeafed, particularly the head of the tibia.
" The patella, with the head of the femur,
" and a portion of the tibia, were removed;
" a good digeftion came on; the limb was
" kept in a ftraight pofition; and on the 21ft
" of November, 1762, he was got fo well as
" to require no farther attention.

" I am extremely forry I cannot give you a
" more particular defcription of this cafe, and
" regret much that my father in his health did
" not inform either you or our worthy friend,
" Dr. Binns, minutely of it. The perfon is
" now living, and fometimes goes to Liver-
" pool, where, if you will give me leave, I
" will defire him to call upon you."

A letter

A letter from Mr. Trye, of Gloucefter, has the following paragraph, which he has obligingly given me his permiffion to infert here :

" Four or five years ago I affifted the late " Mr. Juftamond in removing the olecranon, " and two inches of the ulna, continued from " that procefs, in a man who had a difeafed " elbow joint; and I have lately met with a " boy in whom an accident feparated the os " humeri from its connexion with the bones of " the fore arm, and forced it, denuded of its " periofteum, through the integuments ; I " fawed off two inches and a half of its length, " including the condyles. Both thefe cafes " were completely fuccefsful."

In a fubfequent letter from Mr. Trye, in anfwer to my requeft that he would favour me with farther particulars of the former of thefe cafes, he fays, " The patient, a man, had a " caries in the fuperior extremity of the ulna; " it was not purely fcrophulous, nor were the " ligaments fo much thickened or difeafed as " we commonly find them when a white fwel- " ling has fuppurated and ulcerated : the ole- " cranon, and two inches of the ulna, conti- " nued from it, were removed; to render the " feparating the ulna from its connexion with

"the os humeri more eafy, a portion of the "olecranon was firft chifeled off; the ulna, "cleared from the foft parts, was then fawed "through; the articular furface of the os hu- "meri was not taken off or fcraped away; "the radius was as little meddled with as pof- "fible; nor was there more of the capfular "ligament extirpated than what neceffarily "came away with the portion of ulna: fo "that here the elbow joint was not removed, "but only the extremity of one of the bones "of which it is compounded. The man re- "covered without the leaft untoward fymp- "tom, and had a very ufeful arm; the mo- "tions of flexion and extenfion, as you may "fuppofe, were almoft loft, but he retained "the power of rotating the hand."

The account given of my little pamphlet, in the London Medical Journal, concludes with faying, that, fince its publication, the operation had been repeated with fuccefs, in the elbow, by an ingenious and enterprifing furgeon of one of the London hofpitals. — *Query*, Is not this the cafe I have juft quoted of Mr. Juftamond*? Be that as it may; though Mr. Juftamond's

* Mr. Park is right in his conjecture: it was to the operation

Juſtamond's operation be not a complete extirpation of the joint, it is certainly one of not leſs merit, leſs difficult execution, or leſs happy in its conſequences.

*Liverpool,*
November 5, 1789.

ration performed by Mr. Juſtamond that we alluded. See Vol. IV. of this work, page 282.—Editor.

